# Screening and machine learning-based prediction of translation-enhancing peptides that reduce ribosomal stalling in *Escherichia coli*

**DOI:** 10.1039/d5cb00199d

**Published:** 2025-10-22

**Authors:** Teruyo Ojima-Kato, Gentaro Yokoyama, Hideo Nakano, Michiaki Hamada, Chie Motono

**Affiliations:** a Laboratory of Molecular Biotechnology, Graduate School of Bioagricultural Sciences, Nagoya University Furo-cho, Chikusa-ku Nagoya 464-8601 Japan teruyo@agr.nagoya-u.ac.jp; b Cellular and Molecular Biotechnology Research Institute, National Institute of Advanced Industrial Science and Technology (AIST) Koto-ku Tokyo 135-0064 Japan; c Graduate School of Advanced Science and Engineering, Waseda University Tokyo 169-8555 Japan; d Integrated Research Center for Self-Care Technology (IRC-SCT), National Institute of Advanced Industrial Science and Technology (AIST) Tokyo 135-0064, Japan, Koto-ku Tokyo 135-0064 Japan

## Abstract

We previously reported that the nascent SKIK peptide enhances translation and alleviates ribosomal stalling caused by arrest peptides (APs) such as SecM and polyproline when positioned immediately upstream of the APs in both *Escherichia coli in vivo* and *in vitro* translation systems. In this study, we conducted a comprehensive screening of translation-enhancing peptides (TEPs) using a randomized artificial tetrapeptide library. The screening focused on the ability of the peptides to suppress SecM AP-induced translational stalling in *E. coli* cells. We identified TEPs exhibiting a range of translation-enhancing activities. *In vitro* translation analysis suggested that the fourth amino acid in the tetrapeptide influences the reduction of SecM AP-mediated stalling. Additionally, we developed a machine learning model using a random forest algorithm to predict TEP activity, which showed a strong correlation with experimentally measured activities. These findings provide a compact peptide toolkit and a data-driven approach for alleviating AP-induced ribosome stalling, with potential applications in synthetic biology.

## Abstract 2

Translation-enhancing peptides (TEPs) that reduce ribosomal stallling in *Escherichia coli* were identified through comprehensive screening, and their activity was predicted using a machine learning model.

## Graphical abstract

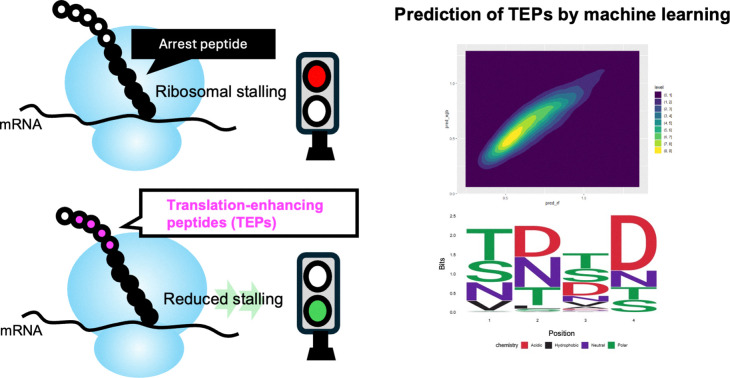

## Introduction

Efficient protein synthesis is crucial for synthetic biology and is increasingly important for sustainable bio-research and industry. However, despite advancements in gene design and codon optimization, the synthesis of proteins of interest (POIs) can be affected by various factors, including promoter strength, the nucleotide sequence of mRNA, and tRNA availability, which can limit protein production yields and compromise the functionality of synthetic circuits.^1^

Translation, a critical step in protein synthesis, is influenced by the sequence of the nascent polypeptide chain itself.^2,3^ Emerging evidence has shown that specific nascent peptide sequences, known as arrest peptides (APs), can interact with the ribosomal exit tunnel, inducing ribosome stalling during elongation.^4–6^ These AP-mediated stalls play pivotal roles in regulatory circuits that modulate gene expression in response to environmental and physiological signals.^3,7^

A notable example is the SecM AP (FSTPVWISQAQGIRAGP) found in *Escherichia coli*, which regulates the translation of secA, an essential component of the Sec protein translocation system.^4^ The SecM AP stalls ribosomes in a sequence-dependent manner, particularly within its arrest motif.^8^ In addition, stretches of consecutive proline residues (polyproline motifs) are known to cause ribosome stalling due to limited prolyl-tRNA availability and slow proline incorporation kinetics.^9^

While these regulatory mechanisms can be biologically advantageous in certain contexts, they present significant challenges in biotechnology and synthetic biology, where efficient and uninterrupted translation is crucial for high-yield protein production. Consequently, overcoming ribosome stalling has become a focal point of research aimed at enhancing recombinant protein expression systems.^10,11^

Our research group previously reported that inserting an “SKIK peptide tag” composed of the four amino acids Ser-Lys-Ile-Lys at the N-terminus of difficult-to-express proteins enhances protein production in both *E. coli in vivo* and *in vitro* systems, as well as in Saccharomyces cerevisiae.^12–14^ This peptide tag has proven effective in increasing protein synthesis, although the underlying mechanism remains unclear.^15–17^ More recently, we showed that the short nascent peptide sequence SKIK, when positioned immediately upstream of an AP like SecM or a polyproline motif, can alleviate ribosome stalling and enhance protein production in *E. coli*.^18,19^ Conversely, Kobo *et al.* reported that AP-induced ribosomal stalling could be mitigated by a selection of randomly chosen tetrapeptides.^20^ Herynek *et al.* employed a more direct screening approach to identify specific N-terminal peptides that enhance soluble production of POIs using GFP-fused constructs and fluorescence-activated cell sorting.^21^ Although the molecular basis for this phenomenon remains largely unexplored, these findings suggest that short peptide sequences might be strategically utilized as translation-enhancing modules in synthetic constructs to boost protein production. However, the specific sequence features that confer translation-enhancing activity are not well understood, and the potential for discovering new translation-enhancing peptides (TEPs) has not been systematically investigated. In synthetic biology, identifying and utilizing TEPs offers a modular and programmable approach to overcoming translation barriers. Integrating TEPs into genetic constructs allows synthetic biologists to fine-tune translational efficiency, optimize metabolic pathway fluxes, and enhance the production of valuable biomolecules such as enzymes, therapeutic proteins, and biomaterials.^22,23^

In recent years, machine-learning techniques have significantly improved our ability to explore protein sequence space.^24–28^ Bayesian optimization frameworks have been utilized to expedite the functional engineering of proteins.^29^ Generative AI models now provide an additional avenue for *de novo* sequence generation.^30–33^ However, both approaches rely on large training datasets and are thus less suitable for designing very short peptides—such as four-amino-acid sequences—where data are inherently scarce.

In this study, we aimed to comprehensively identify novel TEPs capable of alleviating ribosome stalling caused by the SecM AP in *E. coli*. To achieve this, we constructed an artificial randomized tetrapeptide library fused with the SecM AP followed by the superfolder green fluorescent protein (sfGFP) gene. Our screening identified a variety of tetrapeptides with varying strengths of translation-enhancing activity. Furthermore, we applied machine learning methods, including a random forest algorithm, to predict TEP candidates based on sequence features, providing a data-driven strategy for optimizing synthetic biology designs. Our findings yield valuable insights into the design principles governing translation efficiency in the *E. coli* protein expression system.

## Results and discussion

### Screening of TEPs in *E. coli* from the constructed library

**Fig. 1:**
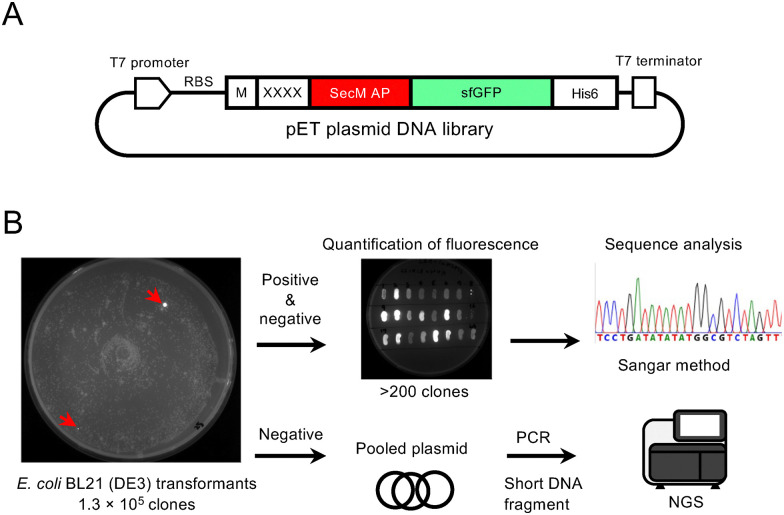
Screening scheme for translation-enhancing peptides in *E. coli*. (A) Plasmid library constructed in this study. RBS, ribosome binding site; XXXX, randomized peptide encoded by (NNK)_4_ sequence. (B) Outline of screening procedure and sequence analysis. Typical culture plates are shown here. Red arrows indicate examples of positive clones displaying fluorescence.

**Fig. 2:**
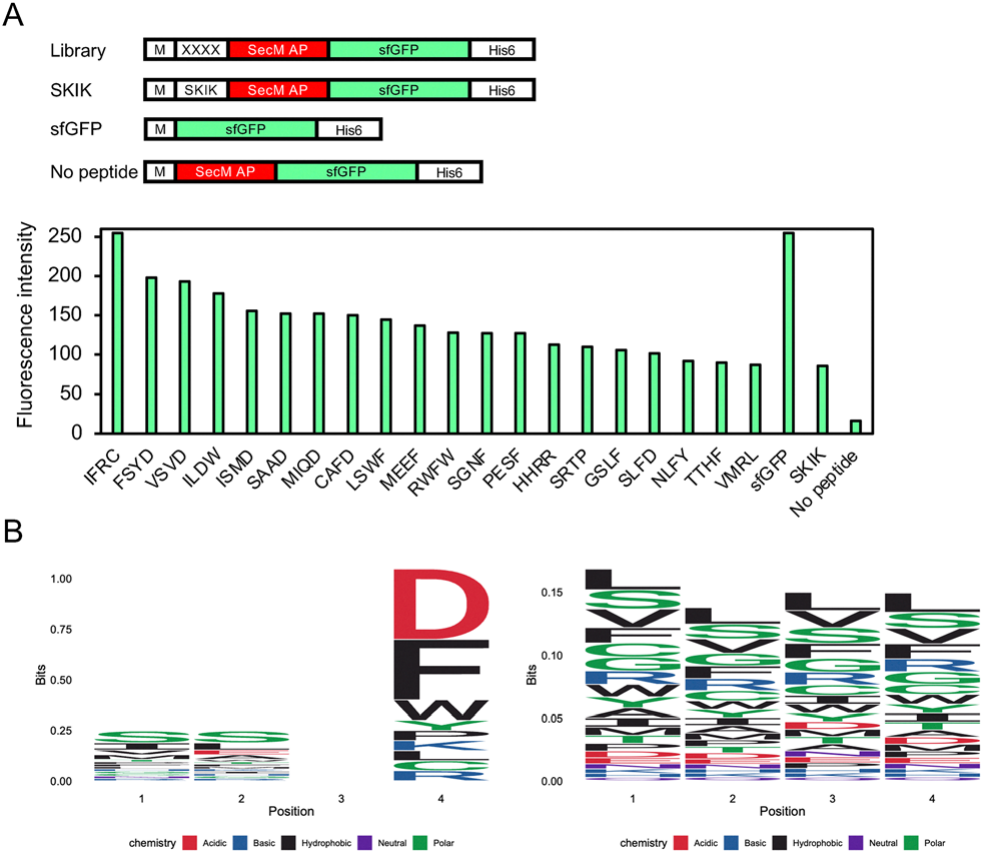
Analysis of the screening result. (A) Relative fluorescence intensity of selected *E. coli*-positive clones on LB agar plates with 1 mM IPTG, exhibiting stronger fluorescence than SKIK. The four-amino-acid sequence corresponds to peptide XXXX in the library plasmid. DNA constructs of samples and controls are illustrated above. (B) Logo plot analysis. Left and right panels show the results of the positive 20 clones exhibiting stronger fluorescence than SKIK and the negative clones analyzed by Nextseq 550, respectively.

Screening was conducted as shown in Fig. 1. We constructed a plasmid library, yielding a total of 1.4 × 10^5^*E. coli* HST08 transformants, and confirmed library diversity by sequencing the randomized (NNK)_4_ positions of several clones (data not shown). The pET22b-(NNK)_4_-SecM AP-sfGFP plasmids extracted from the pooled *E. coli* clones were then used to transform *E. coli* BL21(DE3) for protein expression. Of the total 1.3 × 10^5^ transformants, approximately 0.1% exhibited fluorescence, as shown in Fig. 1(B). Although the total number of screened tetrapeptide sequences did not reach the full 160 000 possible combinations, the scale was sufficient for an *in vivo* screening system. Further analysis of the 217 clones, which included all positive clones with various fluorescence intensities and some clones with lower fluorescence, revealed that they corresponded to 157 unique peptide sequences after removing duplicates, representing a substantial portion of the screened library. The strength of sfGFP fluorescence varied depending on the peptide sequence (Fig. S1). Clones demonstrating higher fluorescence intensity than the previously developed SKIK peptide are summarized in Fig. 2. Notably, no peptide sample without an inserted peptide showed a low intensity value of 16, while that of SKIK was 86. During screening, IFRC exhibited the highest intensity, followed by FSYD, VSVD, ILDW, ISMD, and SAAD. Sequence logos were generated for both positive and negative sequences (Fig. 2(B)). A comparison of two logos indicated that negative clones had a relatively uniform distribution of amino acids at all positions, whereas the positive clones displayed a markedly higher frequency of D at the fourth position, suggesting its potential role in enhancing translation.

### *In vitro* assays using the PURE system

Based on the analysis above, clones exhibiting strong fluorescence—indicative of an effective ability to navigate the ribosomal stalling caused by SecM AP—often had D as the fourth amino acid in the inserted tetrapeptide. Consequently, we experimentally examined any significance of the fourth amino acid on translational enhancement efficiency by substituting FSYD with FSYX (see Table S1). While *in vivo* assays reflect the physiological context, it is difficult to distinguish whether reduced ribosomal stalling is due to the intrinsic effect of the peptide or to cellular rescue factors. To directly assess the contribution of the tetrapeptide on translation, we utilized a reconstituted *E. coli* cell-free translation PURE system which reconstitutes only the minimal components of translation, rather than a live cell expression system.

**Fig. 3:**
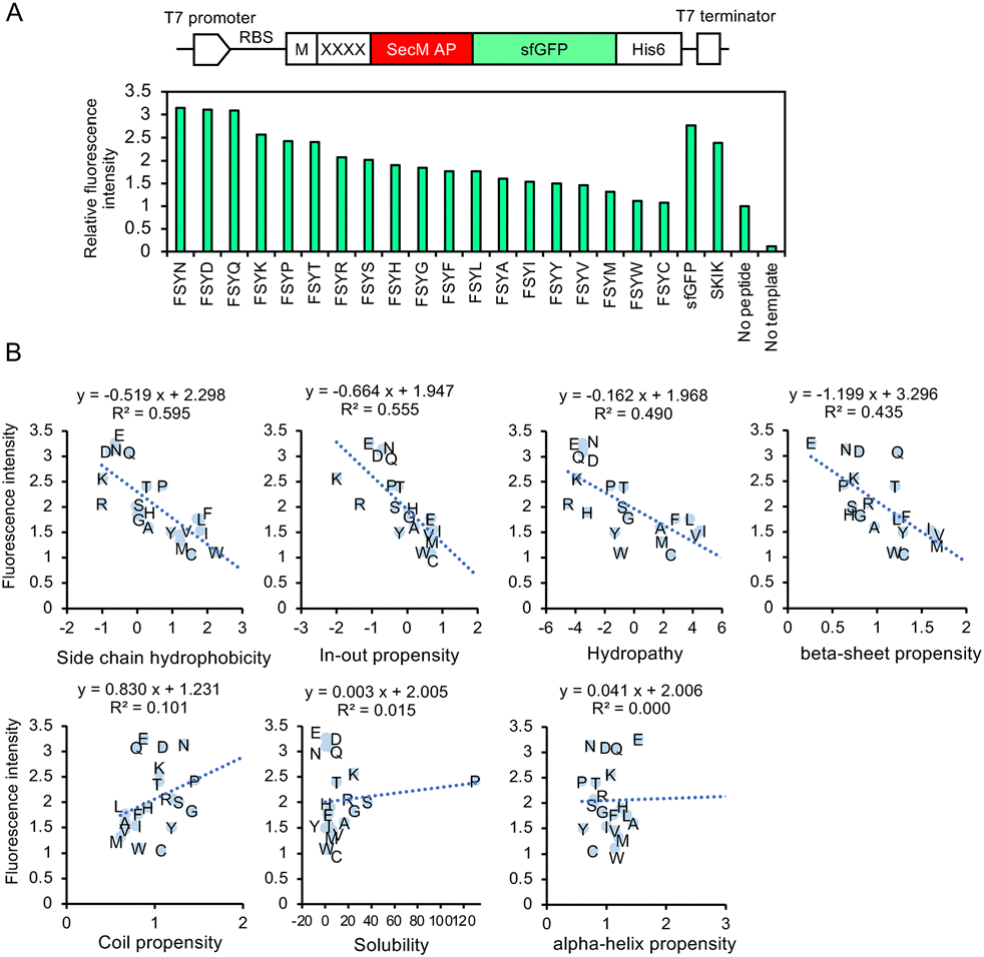
Influence of the fourth amino acid residue in FSYX peptide on alleviating ribosomal stalling by SecM AP. (A) DNA construct used for CFPS and fluorescence analysis. The region XXXX is replaced with the peptide sequence shown here. “No peptide” indicates no XXXX insertion between M and SecM AP. sfGFP serves as a positive control without XXXX-SecM AP. “No template” refers to the negative control of CFPS without any DNA template. The fluorescence intensity of “No peptide” was regarded as 1. (B) Various parameters of the fourth amino acid in FSYX and relative fluorescence intensity. Seven parameters were cited from a study by Nomoto *et al.*^34^

The relative fluorescence intensity of each variant, normalized to 1 for the control without peptide insertion, is displayed in Fig. 3(A). FSYD, FSYE, FYYN, and FSYQ exhibited high fluorescence intensities, followed by FSYK. We then plotted the physicochemical properties^34^ of the fourth amino acid against fluorescence intensity, finding that two parameters—side chain hydrophobicity and in/out propensity—were inversely correlated with fluorescence intensity, with *R*^2^ values exceeding 0.5 (Fig. 3(B)). Interestingly, D, E, N, Q, and K are all classified as polar residues. Notably, with the exception of K, they share the common feature of possessing either a carboxyl or an amide group in their side chains, which may be relevant to their potential contribution to translation-enhancing activity, although any clear association remains uncertain at this stage.

**Fig. 4:**
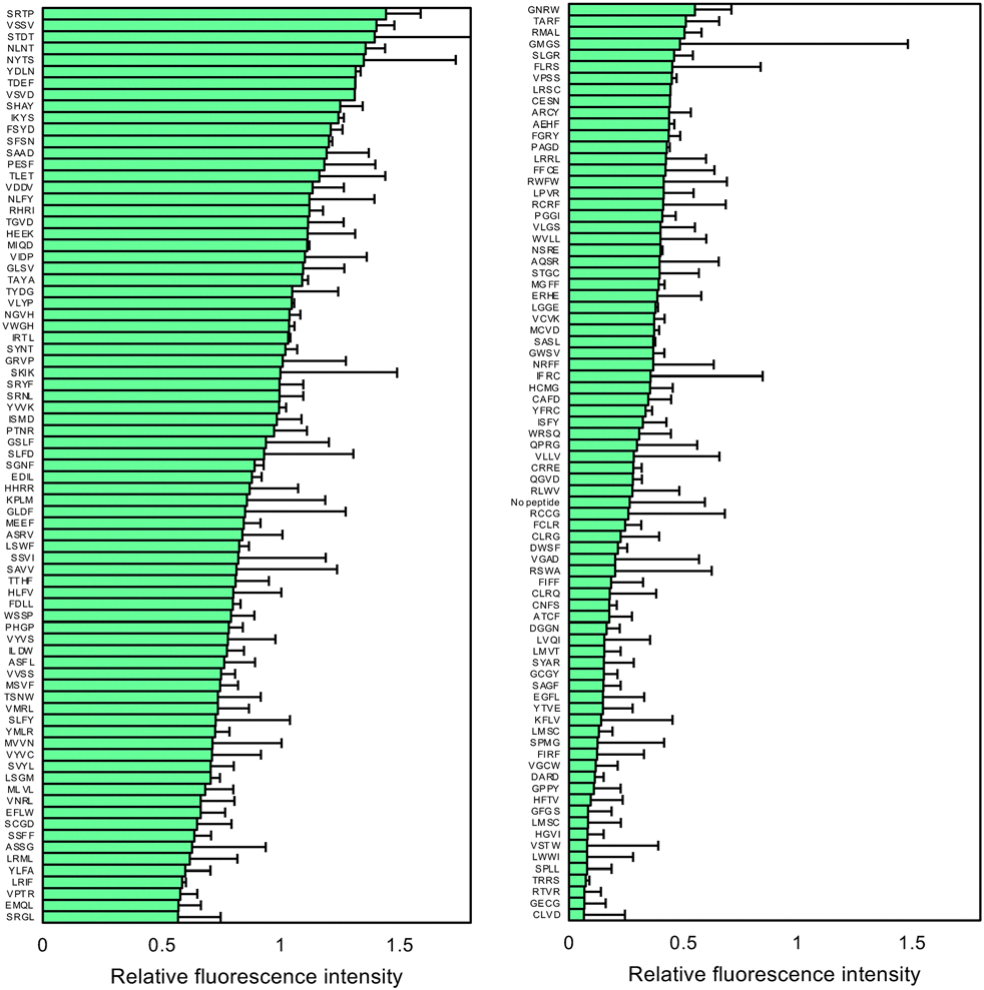
Quantitative fluorescence analysis of the *in vitro* translated products. The positive and randomly selected clones identified through *in vivo* screening were evaluated using CFPS. The relative fluorescence intensities, normalized by SKIK (regarded as 1), are presented. Error bars represent the standard deviation from three independent experiments.

We next evaluated the peptide sequences identified through *in vivo* screening (analyzed by Sanger sequencing) using the PURE system, which is independent of cellular growth conditions and background components. Peptides such as VSVD, FSYD, SAAD, and ISMD—demonstrating high fluorescence intensity *in vivo*—also exhibited strong translation-enhancing effects in the *in vitro* system, with relative values of 1.3, 1.2, 1.2, and 1.0, respectively, when normalized to the translation level of SKIK set at 1.0 (Fig. 4). These findings suggest that while values from *in vivo* and *in vitro* systems do not completely align, many peptides with potential translation-enhancing activity were successfully identified through this screening.

It is well documented that protein expression levels often differ between *in vitro* and *in vivo* systems due to variations in molecular environments, including mRNA stability, folding efficiency, and the presence of regulatory or degradation machinery.^35,36^ Some 5′-UTR sequences enhanced protein expression in *E. coli* strains JM109 and BL21, but these effects were not consistently replicated in a cell-free *in vitro* system,^37^ indicating the difficulty of identifying factors that function universally across both environments.

Trans-translation is recognized as a quality control mechanism in bacteria that rescues stalled ribosomes on defective mRNAs.^38–40^ It involves transfer-messenger RNA (tmRNA) and the protein SmpB, which work together to release the ribosome and tag the incomplete polypeptide for degradation. Notably, the ribosome rescue system differs between *in vivo* and *in vitro* translation. In living *E. coli* cells, *trans*-translation actively resolves ribosome stalling. However, this rescue pathway is absent in reconstituted *in vitro* systems like the PURE system unless tmRNA and SmpB are supplemented. Therefore, translation stalling events can yield different outcomes depending on the system used.

Strategies involving modifications to the N-terminus of the gene of interest or the addition of tetrapeptides to enhance target protein production have been reported.^21,41^ However, to our knowledge, this is the first comprehensive screening to use the apparent alleviation of translation stalling caused by APs as a selection criterion.

### First round bioinformatics analysis

**Fig. 5:**
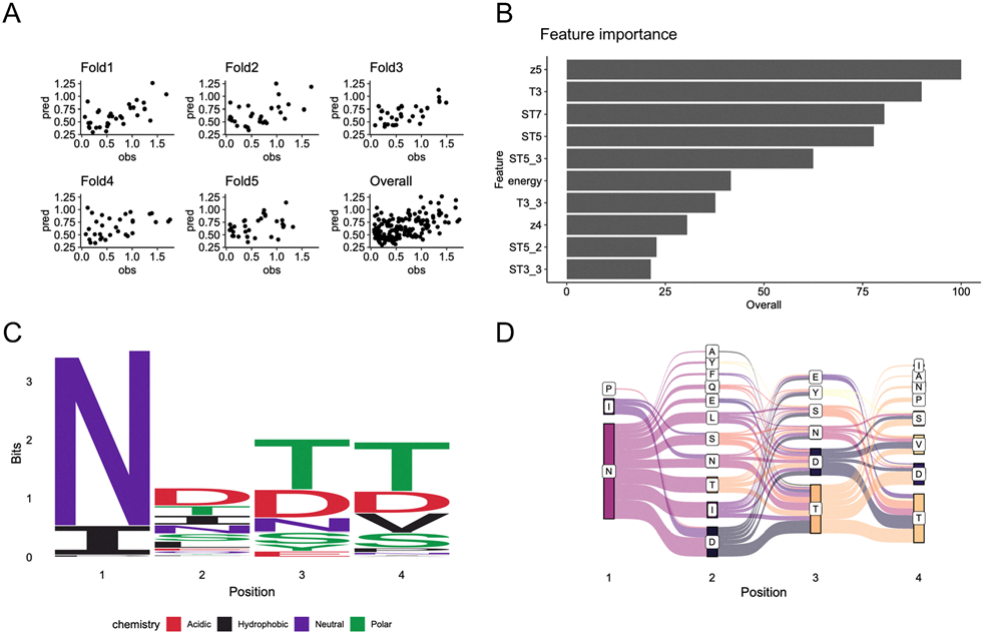
Analysis of *in vitro* data and first prediction of positive peptide sequences. Regression using a 5-fold cross-validated random forest was performed, with the measured relative fluorescence intensity *in vitro* (normalized to a value of 1 for SKIK) as the response variable. The explanatory variables included Z-scale, T-scale, ST-scale, VHSE-scale, and the ensemble energy of mRNA. (A) Scatter plots of predicted values (pred) and observed values (obs) for each fold and across all folds. (B) The top 10 most important explanatory variables in the trained random forest model. (C) Sequence logo of the top 100 peptide sequences with the highest predicted fluorescence values. (D) Sankey diagram of the top 100 peptides with the highest predicted values.

To construct a TEP prediction model, we employed and compared two machine learning methods: random forest^42^ and XGBoost.^43^ While XGBoost generally provides higher accuracy than random forest, it is susceptible to becoming trapped in local minima. In the initial training, we used a dataset of 158 sequences (157 newly designed peptides and SKIK) alongside their *in vitro* translation-derived fluorescence intensities (Fig. 4). Since direct learning from amino acid letters did not yield reliable predictions, we utilized established amino acid descriptors—Z-scale,^44^ T-scale,^45^ ST-scale^46^ VHSE-scale,^47^ and EnsembleEnergy^48^—as explanatory variables. The performance of the first random forest model is summarized in Fig. 5. The Pearson correlation coefficient and root mean square error (RMSE) between predicted and observed values for the overall model were 0.50 and 0.37, respectively (Fig. 5(A)). Feature importance analysis revealed that position-independent descriptors such as Z-scale component 5 and T-scale component 3 were among the most influential features, with EnsembleEnergy variables also ranking highly (Fig. 5(B)). A sequence logo generated from the top 100 predicted sequences with high fluorescence indicated a strong preference for N as the first amino acid residue (Fig. 5(C)). Additionally, a Sankey diagram illustrating patterns of adjacent amino acids revealed a dominant layout of peptide sequences (Fig. 5(D)). The results of the XGBoost model are presented in Fig. S2, showing a Pearson correlation coefficient of 0.51, comparable to that of the random forest model.

### Establishing a loop to improve accuracy of prediction by incorporating new data

**Fig. 6:**
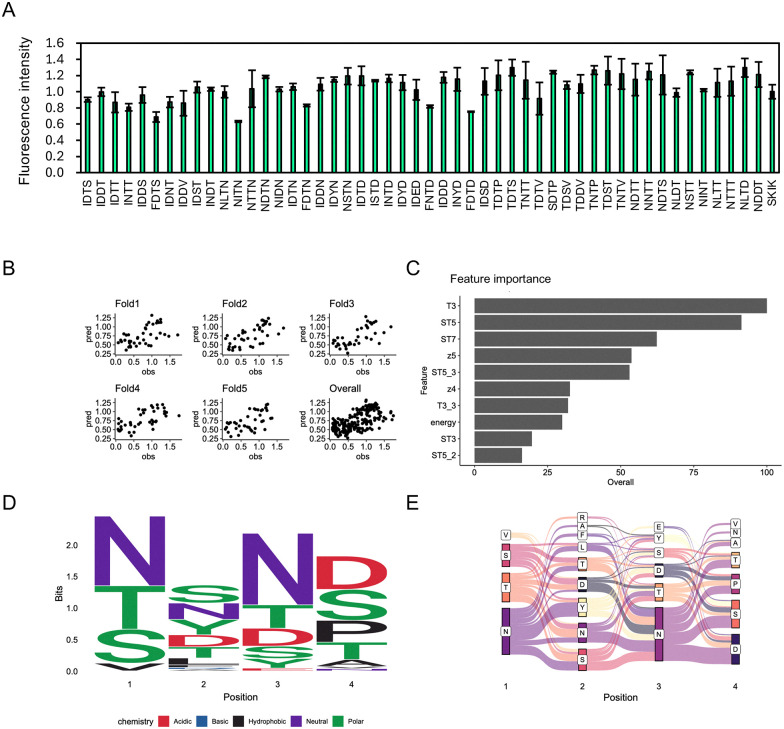
Analysis of *in vitro* data and the second prediction from the random forest model. (A) From the first round of model training, 50 peptide sequences predicted to exhibit high fluorescence intensity were selected. Their translation-enhancing activities were evaluated by measuring sfGFP fluorescence in a CFPS system. Relative fluorescence intensities are shown, using SKIK as the reference standard with a value of 1. The corresponding peptide and DNA sequences are listed in Table S2. (B) Scatter plots of predicted values (pred) and observed values (obs) for each fold and across all folds. (C) Top 10 most important explanatory variables in the random forest model trained with the additional data from panel A. (D) Sequence logo of the top 100 peptide sequences with the highest predicted fluorescence values. (E) Sankey diagram of the top 100 peptide sequences with the highest predicted fluorescence values.

To evaluate the performance of the initial prediction model, we experimentally assessed 50 peptides with the highest predicted fluorescence intensities from a pool of 5000 candidates generated by the first-round random forest training and were experimentally assessed using *E. coli* reconstituted cell-free protein synthesis (CFPS) (Table S2). The results indicated that many of these peptides exhibited translation-enhancing activity comparable to that of SKIK (Fig. 6(A)).

To improve predictive accuracy, a second round of machine learning was conducted using the experimental data from the initially predicted 50 peptides, along with the original dataset of 158 sequences as training data. Following this second-round training, the overall correlation between predicted and experimentally measured fluorescence intensities across all cross-validation folds increased from 0.50 to 0.64 (Fig. 5(A) and 6(B)). Similarly, the correlation coefficient for the XGBoost model improved from 0.51 to 0.63 (Fig. S3). Although random forest and XGBoost do not exactly coincide, they showed the same trend.

The density maps in Fig. S4, which display the predicted fluorescence intensities, indicate that overall fluorescence is distributed primarily at lower intensities. This pattern suggests that highly active TEPs are relatively rare, aligning with the experimental results obtained from the screening.

Sequence logo analysis (Fig. 6(C)) and Sankey diagram visualization (Fig. 6(D)) of the top 100 predicted peptides revealed patterns consistent with those observed in the first round (Fig. 5(C)). Notably, hydrophilic amino acids were favored across all positions, with D frequently enriched at positions 2 to 4.

**Fig. 7:**
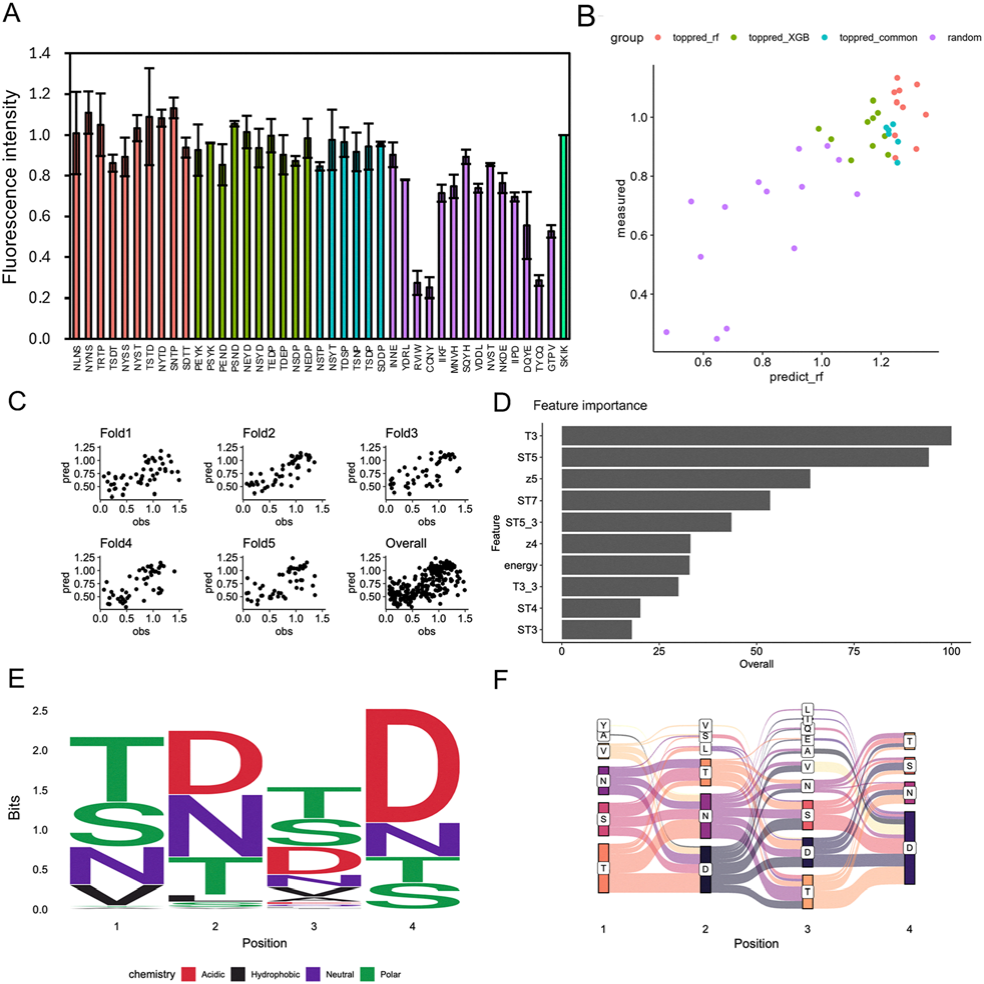
Analysis of various peptides predicted by the second and third predictions from the random forest model. (A) Relative fluorescence intensities of sfGFP expressed in a cell-free protein synthesis (CFPS) system are shown for peptide sequences predicted in the second round of training: the top 10 sequences from the random forest (red), the top 10 from XGBoost (green), the top 10 common to both models (cyan), and 14 randomly selected sequences regardless of predicted intensity (purple). Each peptide was fused to the N-terminus of SecM AP-sfGFP. The fluorescence intensity is relative to SKIK, which has a value of 1, along with mean and standard deviation from triplicate experiments. (B) The relationship between the predicted fluorescence intensities from each of the two trained models and the experimentally measured values shown in panel A is presented. The sample colors correspond to the bar colors in panel A. (C) Scatter plots of predicted values (pred) and observed values (obs) for each fold and across all folds. (D) Top 10 most important explanatory variables in the random forest model trained with additional data from panel A. (E) Sequence logo of the top 100 peptide sequences with the highest predicted fluorescence values from the random forest learning model. (F) Sankey diagram of the top 100 peptide sequences with the highest predicted fluorescence values.

To assess prediction accuracy after the second round of training, we selected the top 10 peptides with the highest predicted fluorescence from both the random forest and XGBoost models. Additionally, 6 peptides that ranked highly in both models and 15 randomly chosen sequences, regardless of predicted values, were included for experimental validation (Table S3). The measured fluorescence intensities are shown in Fig. 7(A), and their comparison with predicted values (Fig. 7(B)) indicates a strong correlation (*R* = 0.83) across both top-ranked and random sequences.

The third round of machine learning utilized the second training dataset, supplemented with the experimentally measured values of the 50 peptides described above. The correlation coefficient improved to 0.66 in the random forest model (Fig. 7(C)) and to 0.65 in the XGBoost model (Fig. S5), showing comparable performance. A summary of model accuracy across all rounds is provided in Table S4.

The sequence logo for the top 100 predicted peptides showed patterns consistent with previous rounds: hydrophilic residues were favored at all positions, with aspartic acid (D) frequently enriched at positions 2–4, particularly at position 4 (Fig. 7(E)). The Sankey diagrams also consistently highlighted preferred dipeptide motifs such as NN, SN, NS, ND, NT, and NP (Fig. 5(C), 6(D), and 7(F)). These consistent patterns in the sequence logos and Sankey diagrams across all three rounds indicate that the models effectively identified common physicochemical features that define TEPs.

### Feature analysis of TEPs predicted by trained models

Across all three rounds of machine learning, the top 10 most important features among the 157 amino acid descriptors remained largely consistent (Fig. 5(B), 6(C) and 7(D)). In the first round, key features associated with translation-enhancing activity included z5, T3, and ST7. In the second and third rounds, T3, ST5, ST7, and z5 consistently ranked among the top contributors. T-Scales summarize 67 topological descriptors related to amino acid connectivity into five principal components, explaining 91.1% of the variance. ST-Scales expand on this by incorporating 827 3D structural features, compressed into eight components that account for 71.5% of the variance. These scales, along with MS-WHIM, are known for their similar behavior in capturing amino acid similarity.^49^ Z-Scales, derived from experimental data such as NMR and thin-layer chromatography, represent properties including lipophilicity (Z1), bulk (Z2), polarity/charge (Z3), and more complex characteristics like electronegativity and electrophilicity (Z4, Z5). While all these scales are derived through principal component analysis, direct interpretation of individual components is often challenging. However, they are widely used to reflect amino acid similarity and behavior in a compact, informative manner.

Across three rounds of random forest modeling, the correlation between predicted and measured fluorescence values steadily improved, indicating enhanced predictive power as training data accumulated. Notably, certain features, such as T3, ST5, and z5, consistently ranked among the most important variables throughout all rounds. This stability in feature selection supports the reliability and robustness of the model, even in the presence of descriptor redundancy.

This study demonstrates that machine learning can effectively predict TEP candidates from a vast sequence space of 160 000 possibilities, utilizing only a small experimentally measured dataset. Starting with a low-bias training set, iterative model updates incorporating predicted high-performing sequences improved accuracy. Given the unknown mechanisms of TEPs and limited data, interpretable models like random forest and XGBoost were well suited to this approach. This strategy efficiently narrowed the search space and identified novel TEPs that could not be discovered through experimental screening alone.

## Experimental

### Construction of the plasmid library

The pET22b-SecM AP-sfGFP plasmid (constructed in our previous study^18^) was used as the PCR template. To insert (NNK) × 4 codons immediately after the initiation codon ATG, inverse PCR was performed using the primer pair 5′-GGAGATATACATATGN̲N̲K̲N̲N̲K̲N̲N̲K̲N̲N̲K̲TTCAGCACGCCCGTCTGGATAAG-3′ and 5′-CATATGTATATCTCCTTCTTAAAGTTAAAC-3′ with KOD One polymerase (Toyobo, Osaka, Japan). The underlined nucleotides correspond to the randomized four amino acids. The PCR product was treated with DpnI (Takara Bio, Kusatsu, Japan, 37 °C for 30 min and 70 °C for 10 min for inactivation) and purified using a spin column (Econospin, Ajinomoto Bio-Pharma, San Diego, CA). The purified linear vector DNA was then self-assembled using Gibson assembly (New England Biolabs, Ipswich, MA). High-performance *E. coli* HST08 competent cells (Takara Bio) were transformed with the product, and colonies were grown on LB plates containing 100 mg L^−1^ ampicillin. The colonies were pooled with TE buffer (10 mM Tris–HCl, 1 mM EDTA, pH 8.0) followed by plasmid extraction using a commercial plasmid purification kit (Plasmid DNA Extraction Midi Kit, Favorgen Biotech Corp., Ping Tung, Taiwan).

### Screening of *E. coli* clones

The constructed plasmid library “pET22b-(NNK)_4_-SecM AP-sfGFP” was introduced into *E. coli* BL21 (DE3) strain for protein expression. The cells were spread on LB agar plates containing 100 mg L^−1^ ampicillin and 1 μM isopropyl β-d-thiogalactopyranoside (IPTG). Competent cells were prepared using a commercially available kit (Mix & Go, Zymoresearch, Irvine, CA). Colonies grown on the LB plates at 37 °C for 16 h were analyzed using a MultiImager II fluorescent imaging apparatus with the appended software MISIS II (BioTools Co., Ltd, Takasaki, Japan; filter set ex 485 nm, em 590/60 nm band-pass, gain setting 13 dB, exposure 16 ms). The picked single colonies were suspended in 50 μL of sterile water and further spotted onto fresh culture plates under the same conditions and incubated for an additional 16 h at 37 °C. The brightness of the fluorescence from the growing colonies was analyzed using ImageJ software.

### Sequencing analysis

Sequencing analysis of all positive clones exhibiting fluorescence and some negative clones with low fluorescence was performed using the Sanger method. Colony-directed PCR products were amplified with the primers F1 (ATCTCGATCCCGCGAAATTAATACG) and R1 (TCCGGATATAGTTCCTCCTTTCAG), which anneal upstream of the T7 promoter and downstream of the T7 terminator, respectively. Remaining *E. coli* clones that did not exhibit significant fluorescence were classified as negative. They were suspended in TE buffer and pooled from the agar plates, and their plasmids were extracted as described above. The DNA fragment (161 bp) containing the randomized region was amplified with the primer pair AAGAAGGAGATATACATATG and ATTAACATCACCATCCAGTTC from the extracted plasmid as the template. The purified DNA fragment was analyzed with the Nextseq 550 using single-end read mode (81 bp) to fully cover the randomized region. The NEBNext Ultra II DNA Library Prep Kit for Illumina and NextSeq 500/550 High Output Kit v2 (75 cycles) were used for sample preparation. The resulting data were analyzed using Seqkit^50^ and a Python program to generate a peptide sequence list of the negative clones.

### *In vitro* protein expression

To confirm the effect of the obtained peptides on translation, cell-free protein synthesis (CFPS) was conducted using PUREfrex 2.1 (GeneFrontier, Kashiwa, Japan) with DNA fragments amplified using Gflex DNA polymerase (Takara) and F1 and R1 primers from single colonies. CFPS conditions were as follows: DNA template; 1 μL, Solution I; 2 μL, Solution II; 0.125 μL, Solution III (ribosome); 0.5 μL, and DEPC-treated RNase-free water (Nacalai Tesque, Kyoto, Japan); 1.375 μL. Reactions were performed at 37 °C for 90 min in triplicate. Each CFPS reaction solution was diluted 25-fold with water, and 50 μL was dispensed into the wells of a Black Microplate Flat Bottom 96-well (Stem, Hino, Japan). The fluorescence intensity of sfGFP was measured using a microplate reader (Infinite 200 PRO, TECAN, ZH, Switzerland) at an excitation wavelength of 485 nm (bandwidth 9 nm) and an emission wavelength of 535 nm (bandwidth 20 nm).

### Construction of the mutants

The peptide FSYX (where X represents 20 amino acids) and other peptide candidates identified using a machine learning prediction tool were introduced *via* PCR using KOD One (Toyobo) and the corresponding primer pairs, as outlined in Table S1. *E. coli* HST08 competent cells were transformed with the *Dpn*I-treated amplified PCR products. Plasmids containing the correct sequences were purified for further experiments.

### Data mining from *in vivo* data

We conducted a computational analysis to identify the characteristics of TEPs obtained from *in vivo* screening. Peptide sequences with identical amino acid compositions were averaged along with their corresponding fluorescence intensity values, forming a single data point. To explore sequence features associated with translation-enhancing activity, we initially analyzed the sequence composition of both positive and negative clones. Sequences were classified as positive if their fluorescence intensity surpassed that of the SKIK control. Sequence logos were created using the ggseqlogo package in R to visualize amino acid preferences at each position.

### Prediction of TEPs with machine learning

We next used *in vitro* experimental data to train models for predicting novel TEP sequences with high fluorescence. All fluorescence values used for training were normalized relative to the SKIK sequence. The input features comprised four amino acid descriptor sets—Z-scale, T-scale, ST-scale, and VHSE-scale—representing the physicochemical and geometric properties of amino acids numerically. For each descriptor, values were calculated for each residue position and the overall sequence average. In addition, to consider codon-level effects on translation, the mRNA free energy of the first 11 codons was calculated using the EnsembleEnergy function from RNAstructure, as described in a previous study.^51^

The Z-scale, T-scale, ST-scale, and VHSE-scale descriptor sets consist of 5, 5, 8, and 8 features, respectively. Each feature set was applied to six aspects of the peptide sequence: the five individual residue positions (including the N-terminal methionine) and the overall sequence average. This yielded a total of 156 amino acid–based features. We also included the EnsembleEnergy value of the first 11 codons as an mRNA-level descriptor, bringing the total number of features used in model training to 157. To enhance clarity given the large number of features, simplified abbreviations are employed throughout this study. For instance, the average value of a descriptor across the entire sequence is labeled as “z1,” while values corresponding to specific positions include a positional suffix, such as “z1_3,” where “3” indicates the position in the peptide sequence. Position numbering starts at 0, with “0” denoting the initial M residue. The descriptor “EnsembleEnergy” is abbreviated simply as “energy.”

We performed a 5-fold cross-validation to ensure the robustness of our analysis. Random forest and XGBoost models were trained using the caret package in R. The ntree parameter in the random forest was set to 1000 to ensure sufficient convergence, while the mtry parameter was determined through grid search techniques. The optimal model was evaluated using predicted and measured RMSEs. Similarly, in XGBoost, the optimal values for eta, max_depth, gamma, colsample_bytree, min_child_weight, subsample, and nrounds were determined through grid search. In each iteration, the model with the lowest RMSE was selected. However, because RMSE values can be influenced by variations in the range or distribution of the training data, Pearson's correlation coefficient was employed to evaluate and compare model performance across iterations. Unlike RMSE, the correlation coefficient is scale-independent and reflects the strength of the linear relationship between predicted and observed values, making it more suitable for comparisons across datasets of varying sizes. All optimal hyperparameter values are presented in Table S5.

After training, we predicted fluorescence intensity for all possible tetrapeptide sequences (approximately 160 000 combinations) and selected TEP candidate sequences. For experimental verification of these predicted peptides, optimal codons were assigned based on the *E. coli* codon adaptation index.^52^ To maximize sequence diversity, candidates were selected not only based on top predicted scores but also through clustering to ensure broad representation of sequence types. Our experimental process employed an iterative training approach, consisting of three rounds of machine learning and experimental validation. In the first round, 50 high-scoring candidate peptides were selected based on the trained model and evaluated through *in vitro* translation. The measured data were then integrated into the training dataset for the second round. An additional 40 peptides were tested experimentally and included in the third round of training. This iterative process aimed to enhance both the accuracy of the predictive models and the efficiency of candidate identification. Model performance was monitored in each round to track improvements in predictive accuracy.

## Conclusions

We identified a diverse array of short TEPs capable of mitigating ribosome stalling induced by the SecM AP in *E. coli*. A machine learning model trained on these data accurately predicted peptide performance, providing a rational framework for designing translation-enhancing sequences. This study not only offers a compact and tunable peptide toolkit for improving translation efficiency but also demonstrates the utility of data-driven approaches in peptide engineering. Future research may extend this strategy to other organisms or arrest motifs, enabling broader applications in synthetic biology and recombinant protein production.

## Author contributions

Conceptualization: T. O. K. and H. N. Data curation: T. O. K., G. Y., M. H., and C. M. Software: G. Y., M. H., and C. M. Investigation: T. O. K. Writing – review & editing: T. O. K., G. H., and C. M. Funding acquisition: T. O. K. and C. M. Supervision: T. O. K.

## Conflicts of interest

The authors declare no conflicts of interest.

## Supplementary Material

CB-007-D5CB00199D-s001

## Data Availability

The data supporting this article are included as part of the supplementary information (SI). The SI includes detailed peptide and DNA sequence data, machine learning model parameters and performance metrics, and additional figures showing experimental results and prediction analyses. See DOI: https://doi.org/10.1039/d5cb00199d. The software used in this study is available from GitHub at: https://github.com/hmdlab/ml-tep.
