# Supplementary material for: Screening and machine learning-based prediction of translation-enhancing peptides that reduce ribosomal stalling in Escherichia coli
Source: RSC Chem Biol. 2025 Oct 22;7(1):58–66. doi: 10.1039/d5cb00199d (PMC12551147; doi:10.1039/d5cb00199d)
Supplement: CB-007-D5CB00199D-s001 [file CB-007-D5CB00199D-s001.pdf]

Supplementary Information (SI) for RSC Chemical Biology.
This journal is © The Royal Society of Chemistry 2025

Supporting Information

Screening and machine learning-based prediction of translation-enhancing peptides that reduce ribosomal stalling in *Escherichia coli*

Teruyo Ojima-Kato[1§*], Gentaro Yokoyama[2,3§], Hideo Nakano[1], Michiaki Hamada[2,3], Chie Motono[2,4]

[§]T.O.K and G.Y. contributed equally to this work

[1]Laboratory of Molecular Biotechnology, Graduate School of Bioagricultural Sciences, Nagoya University, Furo-cho, Chikusa-ku, Nagoya 464-8601, Japan

[2]Cellular and Molecular Biotechnology Research Institute, National Institute of Advanced Industrial Science and Technology (AIST), Koto-ku, Tokyo 135-0064, Japan

[3]Graduate School of Advanced Science and Engineering, Waseda University, Tokyo 169-8555, Japan

[4]Integrated Research Center for Self-Care Technology (IRC-SCT), National Institute of Advanced Industrial Science and Technology (AIST), Tokyo 135-0064, Japan, Koto-ku, Tokyo 135-0064, Japan

*, the person to whom correspondence

https://orcid.org/0000-0001-5290-5176; E-mail: teruyo@agr.nagoya-u.ac.jp

Supplemental Table 1. DNA sequences corresponding to FSYX

Peptide	DNA	Primers used
FSYD	TTTAGTTATGAT	n/a because this sequence was obtained in the screening
FSYW	TTTAGTTATTGG	catATGTTTAGTTATTGGTTCAGCACGCCCGTCTGGATAAGC<br>TGCTGAACCAATAACTAAACATATGTATATCTCCTTCTTAAAG
FSYL	TTTAGTTATTTA	catATGTTTAGTTATTTATTCAGCACGCCCGTCTGGATAAGC<br>TGCTGAATAAATAACTAAACATATGTATATCTCCTTCTTAAAG
FSYR	TTTAGTTATAGA	catATGTTTAGTTATAGATTCAGCACGCCCGTCTGGATAAGC<br>TGCTGAATCTATAACTAAACATATGTATATCTCCTTCTTAAAG
FSYY	TTTAGTTATTAT	TAGTTATTATTTCAGCACGCCCGTCTGGATAAGC<br>TGCTGAAATAATAACTAAACATATGTATATCTCCTTC
FSYC	TTTAGTTATTGT	TAGTTATTGTTTCAGCACGCCCGTCTGGATAAGC<br>TGCTGAAACAATAACTAAACATATGTATATCTCCTTC
FSYE	TTTAGTTATGAA	TAGTTATGAATTCAGCACGCCCGTCTGGATAAGC<br>TGCTGAATTCATAACTAAACATATGTATATCTCCTTC
FSYK	TTTAGTTATAAA	TAGTTATAAATTCAGCACGCCCGTCTGGATAAGC<br>TGCTGAATTTATAACTAAACATATGTATATCTCCTTC
FSYQ	TTTAGTTATCAA	TAGTTATCAATTCAGCACGCCCGTCTGGATAAGC<br>TGCTGAATTGATAACTAAACATATGTATATCTCCTTC
FSYS	TTTAGTTATTCT	TAGTTATTCTTTCAGCACGCCCGTCTGGATAAGC<br>TGCTGAAAGAATAACTAAACATATGTATATCTCCTTC
FSYG	TTTAGTTATGGT	TAGTTATGGTTTCAGCACGCCCGTCTGGATAAGC<br>TGCTGAAACCATAACTAAACATATGTATATCTCCTTC
FSYF	TTTAGTTATTTT	TAGTTATTTTTTCAGCACGCCCGTCTGGATAAGC<br>TGCTGAAAAAATAACTAAACATATGTATATCTCCTTC
FSYH	TTTAGTTATCAT	TAGTTATCATTTCAGCACGCCCGTCTGGATAAGC<br>TGCTGAAATGATAACTAAACATATGTATATCTCCTTC
FSYN	TTTAGTTATAAT	TAGTTATAATTTCAGCACGCCCGTCTGGATAAGC<br>TGCTGAAATTATAACTAAACATATGTATATCTCCTTC
FSYM	TTTAGTTATATG	TAGTTATATGTTCAGCACGCCCGTCTGGATAAGC<br>TGCTGAACATATAACTAAACATATGTATATCTCCTTC
FSYA	TTTAGTTATGCT	TAGTTATGCTTTCAGCACGCCCGTCTGGATAAGC<br>TGCTGAAAGCATAACTAAACATATGTATATCTCCTTC
FSYP	TTTAGTTATCCA	TAGTTATCCATTCAGCACGCCCGTCTGGATAAGC

		TGCTGAATGGATAACTAAACATATGTATATCTCCTTC
FSYT	TTTAGTTATACA	TAGTTATACATTCAGCACGCCCGTCTGGATAAGC<br>TGCTGAATGTATAACTAAACATATGTATATCTCCTTC
FSYV	TTTAGTTATGTT	TAGTTATGTTTTCAGCACGCCCGTCTGGATAAGC<br>TGCTGAAAACATAACTAAACATATGTATATCTCCTTC
FSYI	TTTAGTTATATA	TAGTTATATATTCAGCACGCCCGTCTGGATAAGC<br>TGCTGAATATATAACTAAACATATGTATATCTCCTTC

Supplementary Table 2. Predicted and measured peptides on the first-round training

Peptide	DNA
IDTS	ATCGACACCTCT
IDDT	ATCGACGACACC
IDTT	ATCGACACCACC
INTT	ATCAACACCACC
IDDS	ATCGACGACTCT
FDTS	TTCGACACCTCT
IDNT	ATCGACAACACC
IDDV	ATCGACGACGTT
IDST	ATCGACTCTACC
INDT	ATCAACGACACC
NLTN	AACCTGACCAAC
NITN	AACATCACCAAC
NTTN	AACACCACCAAC
NDTN	AACGACACCAAC
NIDN	AACATCGACAAC
IDTN	ATCGACACCAAC
FDTN	TTCGACACCAAC
IDDN	ATCGACGACAAC
IDYN	ATCGACTACAAC
NSTN	AACTCTACCAAC
IDTD	ATCGACACCGAC
ISTD	ATCTCTACCGAC
INTD	ATCAACACCGAC
IDYD	ATCGACTACGAC
IDED	ATCGACGAAGAC
FNTD	TTCAACACCGAC
IDDD	ATCGACGACGAC
INYD	ATCAACTACGAC
FDTD	TTCGACACCGAC
IDSD	ATCGACTCTGAC
TDTP	ACCGACACCCCG
TDTS	ACCGACACCTCT
TNTT	ACCAACACCACC

TDTV	ACCGACACCGTT
SDTP	TCTGACACCCCG
TDSV	ACCGACTCTGTT
TDDV	ACCGACGACGTT
TNTP	ACCAACACCCCG
TDST	ACCGACTCTACC
TNTV	ACCAACACCGTT
NDTT	AACGACACCACC
NNTT	AACAACACCACC
NDTS	AACGACACCTCT
NLDT	AACCTGGACACC
NSTT	AACTCTACCACC
NINT	AACATCAACACC
NLTT	AACCTGACCACC
NTTT	AACACCACCACC
NLTD	AACCTGACCGAC
NDDT	AACGACGACACC

Supplementary Table 3. Predicted and measured peptides on the second-round training

Peptide	DNA	Prediction
NLNS	AACCTGAACTCT	Highest by random forest
NYNS	AACTACAACTCT	
TRTP	ACCCGTACCCCG	
TSDT	ACCTCTGACACC	
NYSS	AACTACTCTTCT	
NYST	AACTACTCTACC	
TSTD	ACCTCTACCGAC	
NYTD	AACTACACCGAC	
SNTP	TCTAACACCCCG	
SDTT	TCTGACACCACC	
PEYK	CCGGAATACAAA	Highest by random XGBoost
PSYK	CCGTCTTACAAA	
PEND	CCGGAAAACGAC	
PSND	CCGTCTAACGAC	
NEYD	AACGAATACGAC	
NSYD	AACTCTTACGAC	
TEDP	ACCGAAGACCCG	
TDEP	ACCGACGAACCG	
NSDP	AACTCTGACCCG	
NEDP	AACGAAGACCCG	
NSTP	AACTCTACCCCG	Highly ranked by both random forest and XGBoost
NSYT	AACTCTTACACC	
TDSP	ACCGACTCTCCG	
TSNP	ACCTCTAACCCG	
TSDP	ACCTCTGACCCG	
SDDP	TCTGACGACCCG	
INNE	ATCAACAACGAA	Randomly chosen sequences regardless of the predicted values
YDRL	TACGACCGTCTG	
RVIW	CGTGTTATCTGG	
CCNY	TGCTGCAACTAC	
IIKF	ATCATCAAATTC	
MNVH	ATGAACGTTCAC	
SQYH	TCTCAGTACCAC	

VDDL	GTTGACGACCTG
NVST	AACGTTTCTACC
NKDE	AACAAAGACGAA
IIPD	ATCATCCCGGAC
MDQYE	GACCAGTACGAA
TYCQ	ACCTACTGCCAG
GTPV	GGTACCCCGGTT

Supplementary Table 4. Summary of evaluation metrics for each random forest and XGBoost model.

Round	RF_cor	RF_RMSE	XGB_cor	XGB_RMSE
First	0.50	0.37	0.51	0.37
Second	0.64	0.32	0.63	0.33
Third	0.66	0.28	0.65	0.28

Cor represents the Pearson's correlation coefficient, and RMSE denotes the root mean square error between measured and predicted values. RF and XGB mean random forest and XGBoost, respectively.

Supplementary Table 5. Summary of hyperparameter-tuning results from grid search for each model

Round	RF	XGB						
	mtry	nrounds	max_depth	eta	gamma	colsample_bytree	min_child_weight	subsample
First	75	125	3	0.025	0	0.7	1	1
Second	60	200	2	0.025	0	1	1	1
Third	40	125	5	0.025	0	0.4	1	1

To avoid overcomplicating the XGBoost optimization, the parameters gamma, min_child_weight, and subsample were fixed, as they primarily influence learning rate and model generalization.

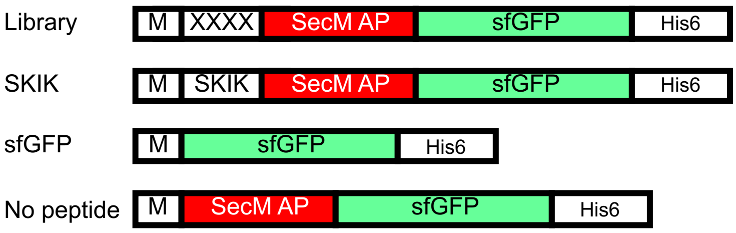

IFRC	255	SRYF	51	RMAL	32	LPVR	15
FSYD	198	YDLN	50	SASL	31	TRRS	15
VSVD	193	YMLR	50	NRFF	31	MLVL	15
ILDW	178	SLGR	49	LGGE	31	GPPY	15
ISMD	156	RSWA	48	ASFL	30	FGRY	15
SAAD	152	SSFF	48	VYVS	30	FFCE	14
MIQD	152	FIFF	47	SFSN	30	TLET	14
CAFD	150	ERHE	47	FCLR	30	VLGS	14
LSWF	145	AEHF	47	TDEF	30	NSRE	14
MEEF	137	PTNR	45	SHAY	30	VLLV	14
RWFW	128	KPLM	45	EDIL	30	YTVE	14
SGNF	127	MSVF	45	LMVT	29	VCVK	14
PESF	127	HEEK	44	TAYA	28	SSVI	14
HHRR	113	CESN	44	VGCW	28	QPRG	13
SRTP	110	VLYP	44	WVLL	27	HGVI	13
GSLF	106	MGFL	43	GRVP	26	VYVC	13
SLFD	102	SRNL	43	GCGY	25	FIRF	13
NLFY	92	FLRS	42	LMSC	25	RTVR	13
TTHF	90	QGVD	42	YLFA	25	SVYL	13
VMRL	87	ASRV	41	TARF	25	CLRG	13
SLFY	81	VGAD	41	SRGL	24	LRRL	13
VDDV	80	DGGN	41	LRML	24	HFTV	12
MVVN	75	EGFL	40	NLNT	24	GFGS	12
VWGH	73	GNRW	40	HLFV	23	DWSF	12
VIDP	73	GLDF	39	EFLW	22	GWSV	12
LWWI	71	NGVH	39	SCGD	22	GECG	12
TGVD	70	PGGI	38	YVVK	21	FDLL	12
NYTS	70	ASSG	38	SAGF	21	VVSS	11
EMQL	68	HCMG	38	STGC	20	RHRI	11
VSSV	67	IKYS	38	STDT	20	SYNT	11
DARD	67	VNRL	37	CNFS	19	SAVV	11
PHGP	65	CLRQ	36	VPTR	18	AQSR	10
RCRF	61	ARCY	35	RLWV	18	ISFY	9
GLSV	61	CLVD	35	VPSS	18	LVQI	9
TYDG	58	CRRE	35	KFLV	17	SPLL	9
TSNW	53	RCCG	35	ATCF	16	PAGD	9
MGFF	53	WRSQ	34	LRSC	16	SPMG	8
WSSP	53	YFRC	33	SYAR	16	sfGFP	255
GMGS	52	MCVD	32	LSGM	16	SKIK	86
LRIF	52	IRTL	32	VSTW	15	No peptide	16

Supplementary Figure 1. Analysis of the effect of four amino acid residues in *E. coli*

Relative fluorescence intensity of the picked *E. coli* clones on LB agar plates with 1 mM IPTG. Positive clones and randomized selected negative ones were analyzed. Four amino acid sequence indicates the corresponding peptide encoded by $(NNK)_4$ in the library plasmid. DNA constructs of samples and controls are illustrated above.

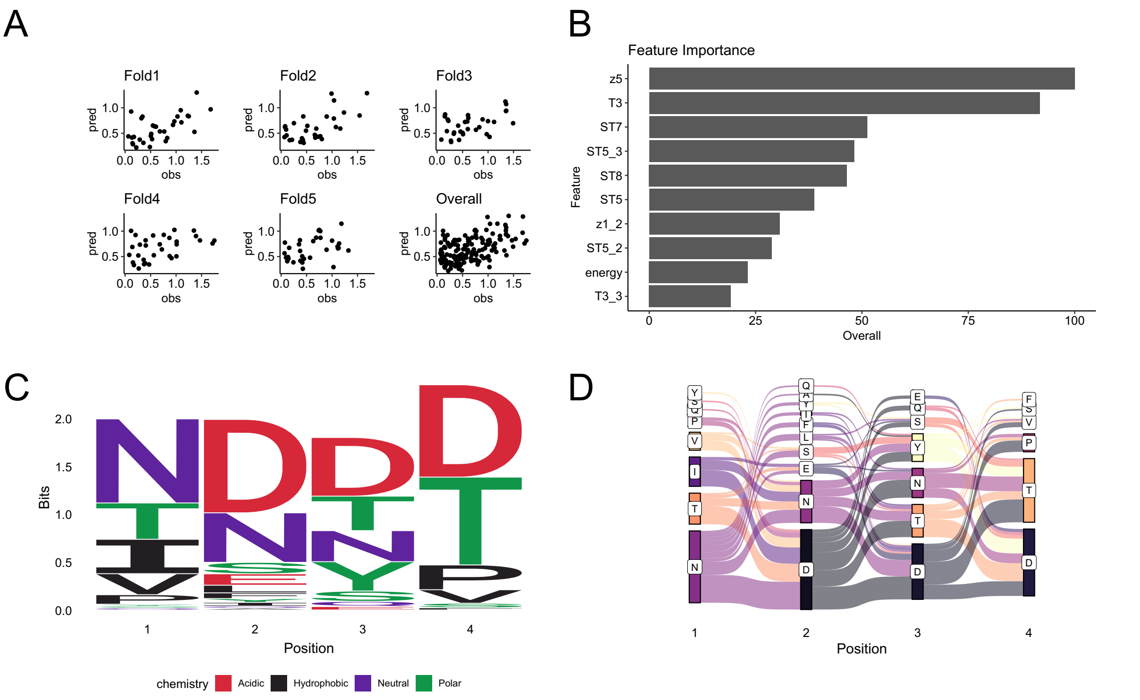

Supplementary Figure 2. Analysis of in vitro data and first prediction of positive peptide sequences by XGBoost model

Regression using 5-fold cross-validated XGBoost was performed, with the measured relative fluorescence intensity in vitro (normalized to a value of 1 for SKIK) as the response variable.

The explanatory variables included z-scale, t-scale, st-scale, vhse-scale, and the ensemble energy of mRNA.

A. Scatter plots of predicted values (pred) and observed values (obs) for each fold and across all folds.

B. Top 10 most important explanatory variables in the trained random forest model

C. Sequence logo of the top 100 peptide sequences with the highest predicted brightness values

D. Sankey diagram of the top 100 peptides with highest predicted values

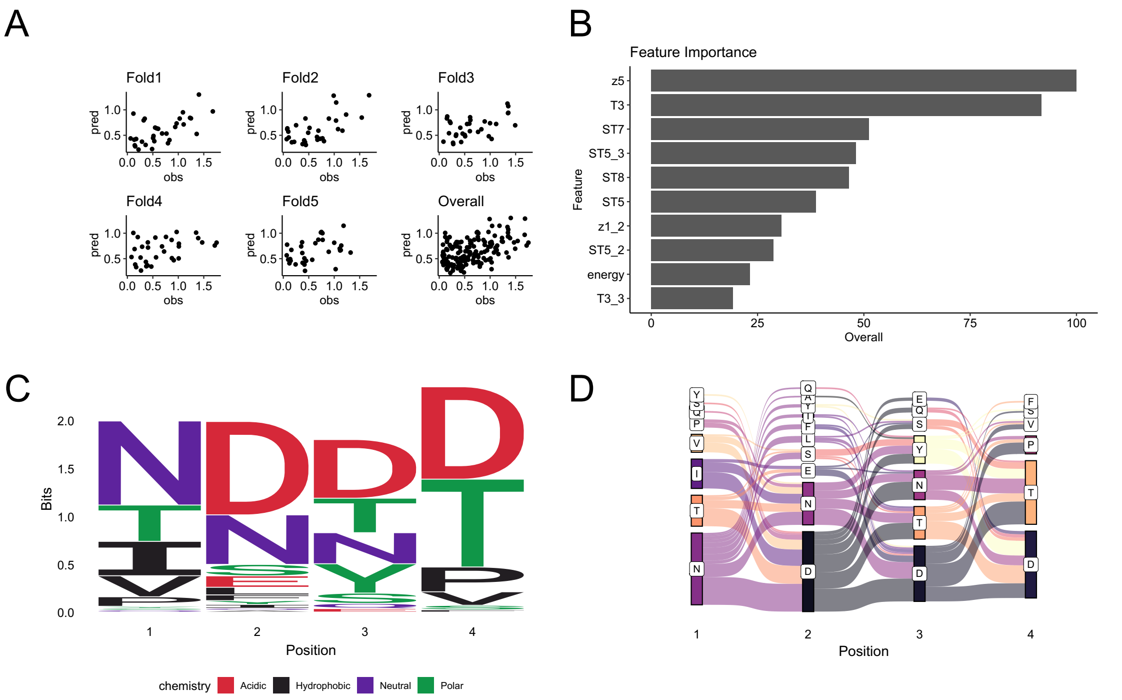

Supplementary Figure 3. Analysis of in vitro data and second prediction by XGBoost model

A. Scatter plots of predicted values (pred) and observed values (obs) for each fold and all folds.

B. Top 10 most important explanatory variables in the XGBoost model trained with the additional data from panel A.

C. Sequence logo of the top 100 peptide sequences with the highest predicted fluorescence values.

D. Sankey diagram of the top 100 peptide sequences with the highest predicted fluorescence values.

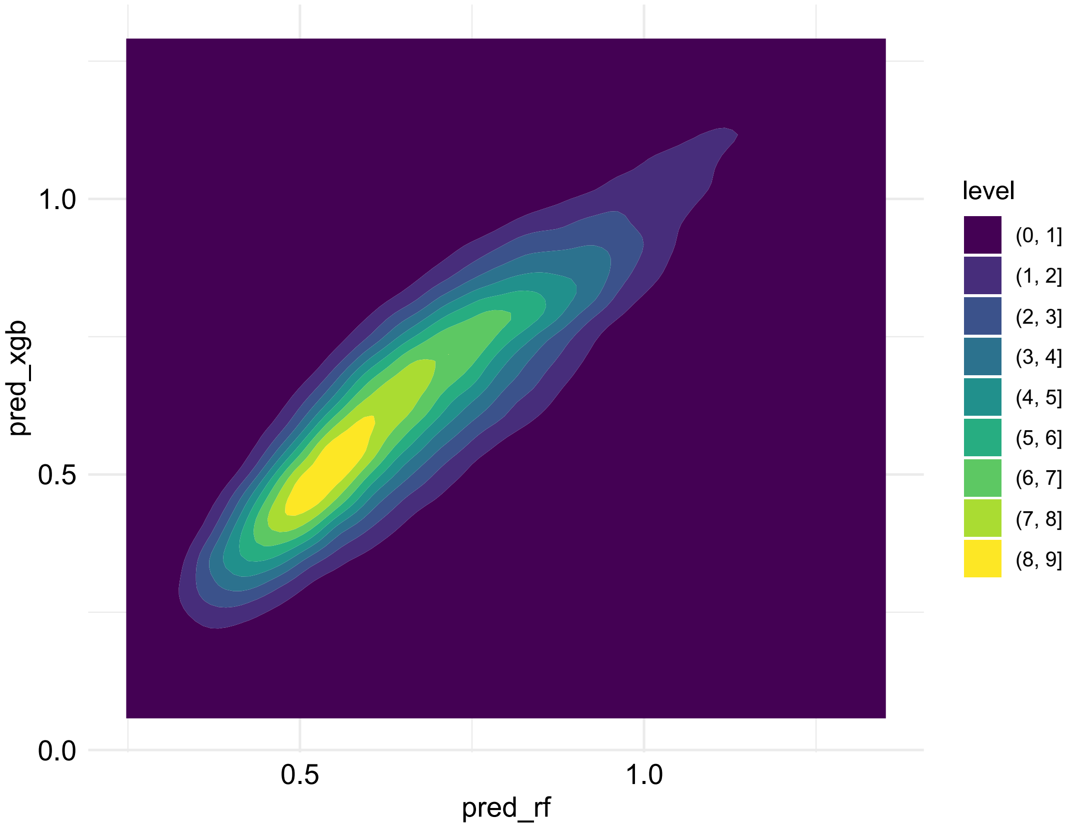

Supplementary Figure 4. Contour plot comparing the predictions of both random forest and XGBoost from the second prediction

Predicted values for all 160,000 possible tetrapeptides excluding measured ones are shown as a 2D kernel density plot, normalized to SKIK = 1. Color indicates density levels. Distributions predicted by random forest (Pred_rf) and XGBoost (Pred_xgb) are shown.

A

B

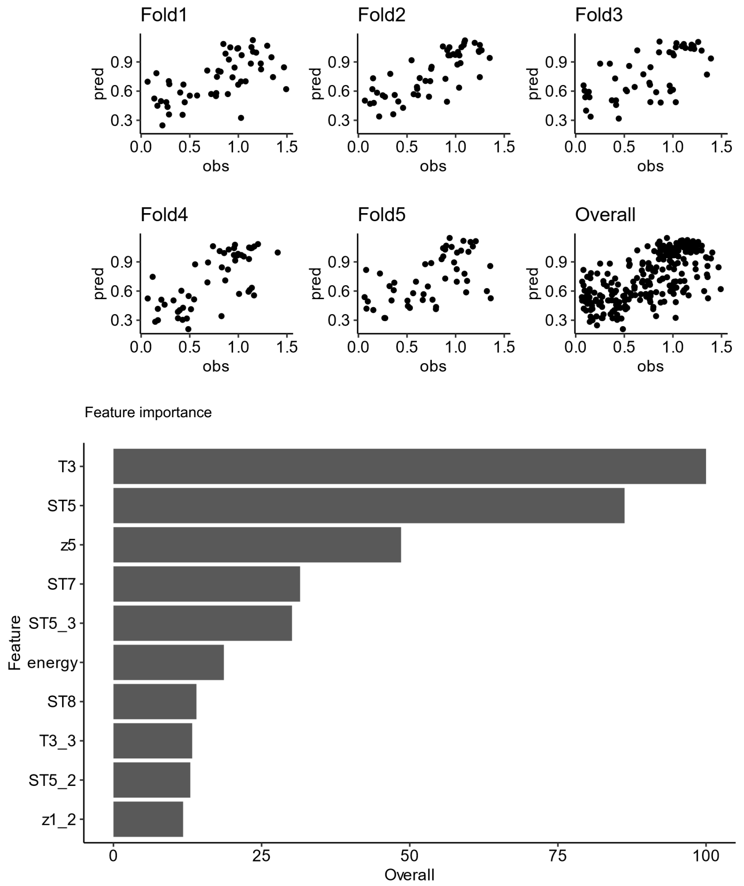

Supplementary Figure 5. Analysis of in vitro data and third prediction by XGBoost model

A. Scatter plots of predicted values (pred) and observed values (obs) for each fold and all folds.

B. Top 10 most important features identified in the XGBoost model trained with the expanded dataset.
